# The Personal Equation in the Use of the Forceps

**Published:** 1896-07

**Authors:** Eugene R. Corson

**Affiliations:** Savannah, Ga.


### Southern Medical Record.

A MONTHLY JOURNAL OF MEDICINE AND SURGERY.

VOL. XXVI. ATLANTA, GA., JULY, 1896. No. 7.

### Original Articles.

### THE PERSONAL EQUATION IN THE USE OF THE FORCEPS.

BY EUGENE R. CORSON, B. S., M. D., SAVANNAH, GA,

(Read by title before the Georgia Medical Association, April 1, 1896.)

My object in presenting this paper is to plead for the intelligent and skillful use of the forceps, and to show certain fallacies in the arguments of those who decry its use. From the beginning of my professional work it has been my lot to get many cases of difficult labor, and I came early to look upon this instrument as one of the most useful in our *armamentarium* and as a great blessing to many suffering women. Its application never seemed to me to be a difficult thing to learn, nor have I found any difficulty in deciding when to use it or to map out in my own mind a definite scheme of the indications which call for it.

I have always felt indebted to the teachings of Robert Barnes, whose "Obstetrical Operations," published over twenty years ago, gave me the clearest idea of the management of difficult labor. This book, I am sure, must be placed among the classics in our literature, and notwithstanding the immense strides which obstetrics has made in the last twenty-five years, studies which have kept step with the advances in the whole domain of surgery and medicine, this book can be read to-day with immense profit by student and practitioner. I am glad to see that a little book on "Difficult Labor," by G. Ernest Herman, a concise and true epitome of the present state of obstetrics, draws largely from the "Obstetrical Operations." And with the exception of the larger field of Cæsarean section, due to improved surgical technique and antiseptics, and to the establishment of the question of symphysiotomy, whose application has been so thoroughly tested by the French school, the general indications for the forceps are practically the same to-day as they were when Barnes wrote his book. To be sure, we know better the dangers of the forceps when misapplied, we know better the evil results which follow this misapplication, and we know better how to guard against the all-important sepsis. We have, perhaps, a slightly better model, and we have the principle of the axis traction introduced by Tarnier, and yet the large majority of our cases fall under the general indications laid down twenty-five years ago.

It would be idle indeed for me to lay down these general indications as they are accepted to-day; they are plainly and succintly set forth in our text-books on the subjects. I contend, however, that these indications are really the indications in the abstract that they must vary more or less according to the skill and ability of the operator, and that, therefore, the utility and value of the forceps is a variable X depending upon the operator. The exact conditions in each case can never be exactly known or predicted. The operator, to know the problem at the time, must not only know the conditions of his patient, but he must know his own fitness or unfitness for operating. The patient remaining the same, the one case may call for an operation, while the other contraindicated it. In attempting to lay down these definite indications I find the authorities omit this important factor in the consideration of the case, this question of the operator himself. Take any tool, and the more skillfully it is handled the more useful and efficient it becomes. An ordinary jack-knife in the hands of a Swiss carver changes a block of wood into a work of art, while the same instrument in the hands of the ordinary boy, brings blood and pain. A good operator with a few instruments accomplishes more than a poor one with any amount of surgical *armamentarium*. Therefore, I say, the important question is, can you use the forceps properly, rather than, are they indicated in this case?—for in the present state of your knowledge of obstetrics, the usual indications are clearly and precisely laid down and are easily enough learned, while one's individual skill must be acquired and consciously realized. And this is usually the last thing considered. Given a case with certain conditions and a good operator, and I should say, operate, while the same case with a poor operator, I should say wait. A case of stone had better wait a reasonable time for a good operator than be cut at once by one who knows not his work. While "Ventral Suspension of the Uterus" for malposition may be a legitimate operation in the hands of an operator like Howard Kelly, who has just reported a series of two hundred case-without a death, the same operation in the hands of a tyro, or one unfitted for any operative work, is contraindicated. Now the same reasoning applies, *mutatis mutandis*, to the forceps.

I have heard an obstetric teacher take every opportunity to discredit the forceps, and when the time came for him to show its application to his class, apply it improperly. Verily, from his standpoint the man was right, for he should never apply it himself unless there was absolutely no other remedy.

I have recently read a paper in the transactions of this Association which has a direct bearing on this point. A physician reports results in five hundred and ninety cases of labor where the forceps was not once applied, and who regrets its non-use but in one case, where he thinks the child might have been saved. Now there is much in that paper which I admire, the man's honesty, than which there is no greater virtue, his conscientiousness, a virtue equally as great, and his courage, too and yet I believe he does not know how to use the forceps, and that he has a subconscious sense of his incapacity. That out of these five hundred and ninety cases all the women were safely delivered simply proves that he practiced in a healthy locality, that his patients were good breeders, and that unaided nature can often work wonders. And I further contend, that had he had the most approved model of the forceps, and not left it at home, as he affirms, and had he had a conscious skill in its use, he might have saved some of these women many hours of suffering, and not only without any injury to them, but with a distinct gain, and he might have saved the only still-born child to which he proudly points. His paper is surely a very significant one.

One of the glories of modern medicine is its power to relieve pain. Diseases which still defy a reduction in their death rate may at least be made less painful and more bearable. Drugs which cannot directly cure or shorten certain diseases can at least make the patient more comfortable. I contend that it is a legitimate function of the forceps to shorten a delivery and to relieve a patient of pain, and that it can be so used without injury to mother or child. That this use of the forceps, outside the domain of absolute necessity, demands greater judgment and discrimination and, I may say, greater skill, I admit, for from its large field of application it is more liable in unskilled hands to do injury. Therefore, I say, the use of the forceps depends as much upon the skill and discrimination of the operator as upon the absolute necessities of the case. Given a labor in any way prolonged, the more skilled you are in the use of the forceps, the better you know the patient, the more certain you are of the position of the child and the condition of the uterus and pelvis, the more unjustified you are in the use of instruments. And, on the contrary, if you are not skilled in its use, if its application simply means to you to stick it in, to lock it, and to pull, you had better sit down by your patients and wait until you are assured there is no other remedy. The proper use of the forceps does not seem to me to be a difficult thing to learn, if one has any mechanical turn at all or manual dexterity, and yet, I have seen it very bunglingly applied, just as I have seen such a simple thing as a hypodermatic injection badly done. Those practitioners who are content to calmly wait and wait, hour after hour, beyond the usual limit of labor, are usually lacking in diagnostic acumen and decision, and have a conscious or subconscious sense of lack of skill in all instrumental work. They wait till uterine inertia sets in, which they try to combat by old women's remedies, or worse still, by ergot; they are always loud in their denunciation of the forceps, and only see the ill effects of instruments misapplied or contraindicated.

I have heard a physician and an obstetric teacher boast of how he had advised in a certain difficult case, against the use of the forceps where other physicians in the case had wanted to use it, but that he had stood firm, assuring them that though the woman was suffering and had already been much longer in labor than the average, and that though she would probably still have several hours of suffering, he was convinced she was equal to the occasion and would eventually be safely delivered. Looking around with a proud smile, he continued, "In eight hours the baby came, and mother and child did well. And, gentlemen, there was no ruptured perineum to sew up, and no lacerated cervix with its subinvolution or endometritis and all its train of ills to keep our patient a wretched invalid."

Before a class of young students this will bring an applause, but before a body of intelligent practitioners it must create a smile. The fallacy of it all is that he takes it for granted that the cervix and perineum will be ruptured. In other words he assumes that the forceps will be improperly used. So far as the perineum goes the forceps has nothing to do with it, for they should be removed before the head is born, and as to the cervix, will any intelligent or experienced physician deny that, in an ordinary case of delayed labor, the forceps can be used without injury to mother or child?

I have given this case because it typifies a class of practitioners who cry out not only against the forceps, but against every other instrument or instrumental method, and who base their opposition upon the misuse or abuse of an instrument. It simply resolves itself into this: Could they themselves use the instrument skillfully they would see the case in a different light.

I must admit to a fairly free use of the forceps and its blessings seem to me greater every day. I have used it when I considered it absolutely necessary, and with equal satisfaction when I felt I had saved the woman an hour or more severe pain, and not only have I failed to see where it has injured either mother or child, but have been convinced it was a blessing to both.

As I have indicated, there are forceps cases and forceps cases, cases which not only demand great skill and a large expend ture of force, but cases where the operation becomes one of the simplest imaginable, cases requiring but little force and but a few minutes to accomplish. It is not much more than giving an enema.

To one in full obstetric practice, how common it is to get cases where the head comes safely through to the pelvic floor and there rests, the uterus apparently satisfied to have brought the labor to that point with no concern for completing it. The pains are short and inefficient, and really distress the patient as much as more efficient ones, while the pressure of the head is very distressing, and the patient becomes irritable and worn out. Forceps here applied require but the gentlest force to bring the head to the vulva outlet, and, unless the operator is a miserable bungler, it is impossible to do harm in any way. This little operation saves perhaps one, two, or three hours,and what a satisfaction to all concerned! Here is a case in point this very day of writing: A woman, healthy and strong, mother of three children, all of whom I delivered easily without any interference, is in her fourth labor. Even in her first confinement the second stage was not over an hour, while the second and third child were delivered even more easily. In this labor pains started at 2:30 A. M., and when seen by me at 10 A. M. I found the child L. O. I. Ant., the cervix fully dilated and the head well engaged and resting on the pelvic floor. Eleven o'clock passed and twelve o'clock with little or no progress; one o'clock passed, and still no progress and the pains worrying and ineffective; half-past one came and no progress and the woman tired and irritable. She had been three and a half hours in the second stage. I now give a little chloroform, apply the forceps without changing her position in bed, and with the slightest force bring the head down till it bulges the perineum; remove the blades and easily delivered the head. The child had a large head and weighed eleven pounds, which explained the delay. I was convinced in this case that I did mother and child not the slightest injury, and I probably saved her one or more hours of suffering. It is hardly a forceps case. And how many such cases come to the obstetrician, and how easily they are relieved! And how many hours of suffering in the aggregate have been saved! Surely such interference is hardly more than relieving an overloaded rectum.

I am fully aware of the dangers of meddlesome midwifery, and especially meddlesome midwifery in ignorant hands, for there is a distinction, but I believe that the ill effects of obstetrical nihilism are equally as great. We, in the South, where there is a large colored element, have abundant opportunities of seeing the results of a *laissez faire* policy as practiced by the ignorant colored midwives upon whose mercy the large majority of the colored throw themselves. My note-book is full of such cases where I have been called in after many hours and even days have gone by, death resulting to both mother and child. A case like the following seems hardly credible in this nineteenth century.

A negress, age sixteen, had been in hard labor three days, the decomposed head of a large child protruded through the vulva; the mother was dying. On traction the head came off from the body; both arms came away in removing the trunk, with the abdomen greatly distended from decomposition; the woman died a few hours later.

This, of course, is an exceptional case, but it shows to what extent this apathy may be carried, and it teaches its lesson.

Here is another case. Called to a woman after five days of hard labor, with, of course, complete uterine inertia. It took me just one-half hour to lock my forceps. I delivered a large dead child with the largest caput succedaneum I have ever seen. The mother recovered. Where but among the colored, except, perhaps, in the wilderness, could one find these results of delay and neglect.

My note-book is full of cases where I have come in after death, cases of eclampsia, a disease to which the colored are susceptible, placenta prævia, occipito posterior position, neglected shoulder presentation, and uterine fibroids obstructing labor.

I can hardly imagine the possibility of vesico-vaginal fistula from the use of the forceps, the cases which come to us now are neglected cases from the country; the more general and intelligent use of the forceps is making this condition rarer and rarer.

The cervical tear so much dwelt upon, and rightly, too, in many cases, depends upon when and how the forceps is used.

There are cases where delivery must be rapid to save either mother or child or both, and where the cervical tear is almost nothing in comparison with the other alternative. When one can wait till the cervix is perfectly dilated, the forceps, skillfully used, tear nothing.

I am glad to quote Munde in this connection, who writes: "The forceps is an eminently conservative instrument, and can never do harm when applied and used under proper conditions, and with a correct understanding of the anatomy of the genital canal and the mechanism of parturition."

This is undoubtedly the opinion of our prominent obstetritians, if we may judge from their writings.

As I have more than once asserted in this paper, the evil results of an instrument improperly used is no argument against that instrument, it should be on the contrary an incentive to its skillful use. Physicians who have to do obstetrical work should strive to attain to that diagnostic and manipulative skill which sanctions the use of instruments. The fear of the forceps must go; only those on the outskirts are afraid of it.

The physician who knows his work, who can make his diagnosis, who understands the cervix in labor, and the limits of the uterine *vis a tergo*, and who appreciates the satisfaction of relieving useless hours of suffering, fears nothing from an instrument which has stood the test of two hundred and fifty years.

